# Identification of SNPs and Candidate Genes Associated with Major Drought Tolerance QTL on Wheat Chromosome 4A

**DOI:** 10.3390/plants15060921

**Published:** 2026-03-16

**Authors:** Joanne Caguiat, Md Sultan Mia, Hui Liu, Guijun Yan, Kadambot H. M. Siddique

**Affiliations:** 1 UWA School of Agriculture and Environment, The UWA Institute of Agriculture, The University of Western Australia, Perth, WA 6009, Australia; hui.liu@uwa.edu.au (H.L.); guijun.yan@uwa.edu.au (G.Y.); kadambot.siddique@uwa.edu.au (K.H.M.S.); 2 Plant Breeding and Biotechnology Division, Philippine Rice Research Institute, Science City of Muñoz, Nueva Ecija 3119, Philippines

**Keywords:** chromosome 4A, drought, grain yield, near-isogenic lines, 90K SNP, wheat (*Triticum aestivum* L.)

## Abstract

Wheat (*Triticum aestivum* L.) is one of the most cultivated crops in the world, but production is often affected by drought. The wheat chromosome 4A contains several quantitative trait loci (QTL) associated with drought tolerance and yield-related traits, making it a valuable target for genetic improvement. In this study, we developed near-isogenic lines (NILs) carrying *qDT.4A.1*, a major QTL for yield using a fast generation cycling system (FGCS) and characterized these NILs for grain yield and thousand-grain weight (TGW) under drought stress and control conditions. We identified a single nucleotide polymorphism (SNP) marker Kukri_c27037_112, which showed a consistent genotype–phenotype associations across two NIL pairs. This marker is linked to four candidate genes encoding a RING-finger E3 ubiquitin ligase, a receptor kinase, and a protein kinase family protein involved in drought stress response and pathways. In silico expression analyses revealed upregulation of these genes in grain tissue under drought conditions, supporting their potential role in grain development and yield formation during drought stress conditions. The identified SNP marker and its associated candidate genes are potential resources in marker-assisted selection and fine mapping pending further validation and functional studies. Our results provide valuable genomic resources, laying the foundation for the development of drought-tolerant wheat varieties and highlighting chromosome 4A as a key region governing drought tolerance.

## 1. Introduction

Wheat (*Triticum aestivum* L.) is an important cereal crop globally, providing over 20% of the world’s calorie intake [1]. In 2022, global wheat production reached 778 million tons [2]. However, climate change poses a significant threat to wheat production [3], with drought stress affecting approximately 60% of wheat-growing regions worldwide. Drought conditions adversely impact essential physiological processes in plants, leading to reduced growth, yield losses, and compromised crop quality [4,5]. Identifying drought-tolerant genetic traits and understanding their expression patterns under drought stress are essential for developing drought-tolerant wheat [6]. Research shows that breeding drought-tolerant wheat varieties and understanding stress tolerance mechanisms will help increase wheat production and maintain yield stability amidst climate change. Successful breeding programs require an integrated approach that combines phenotypic and genotypic traits [7]. Quantitative trait locus (QTL) mapping is an essential tool in identifying genomic regions associated with traits of interest such as drought tolerance. This process involves detecting QTL for target traits, narrowing down interval regions, and locating tightly linked markers for marker-assisted selection [8,9]. However, the direct use of QTL markers in breeding programs remains challenging due to the large genomic intervals of most reported QTL [10]. To overcome this limitation, developing and utilizing near-isogenic lines (NILs) is an effective approach [11]. NILs have a genetic background identical to that of isolines, except for a targeted single locus [12]. Evaluating the phenotypic effects associated with a particular QTL of the gene through characterization of the morphological and phenological characteristics of NIL plants can help identify candidate genes for the studied traits [13]. Genotype-phenotype association analysis has proven effective in candidate gene identification [10,14,15,16] and, consequently, can be used in identifying single nucleotide polymorphisms (SNPs) for Kompetitive Allele Specific PCR (KASP) conversion [17].

Several studies have identified QTLs and genes associated with drought tolerance across different wheat chromosome regions [18,19,20,21,22]. Among these regions, chromosome 4A emerged as a particularly important key region due to several and consistent QTLs linked with drought-related traits identified across diverse genetic backgrounds and environments. This chromosome region has been reported to contain several stable QTLs associated with yield, stress indices, and water-use efficiency under drought conditions. For instance, a significant QTL for drought susceptibility index, located near marker Xwmc89 on 4A, explained 41% of the phenotypic variation from the Dharwar Dry parent [22]. Another study reported a QTL (*QGyp.ksu-4A*) for grain yield under drought conditions on chromosome 4A that explained 16.3% of the phenotypic variance [23]. Additionally, markers predominantly located on chromosome 4A, including one associated with a drought tolerance index, explained 6% of the phenotypic variation and were stable across multiple field environments [24]. Similarly, the Babax variety harbors a major effect on QTL for yield under drought stress, explaining 27% of the phenotypic variation [20]. Chromosome 4A also harbors important yield-related traits. NILs derived from a Babax x Dharwar Dry population have been characterized via 90K SNP genotyping, identifying a significant 4A QTL at 0.14 Mb between markers Xgwm397 and Xwmc491 that is associated with biomass per plant and grain number [15]. This region overlaps with other reported QTLs for plant height, yield, and biomass [25,26,27]. A genotype–phenotype association study using NILs revealed candidate genes linked to root traits in the *qDT.4A.1* genomic region [14]. A multi-environment QTL analysis of Seri × Babax recombinant inbred lines (RILs) further revealed two stable QTLs on chromosome 4A, associated with grain yield and grain number, explaining 14.8% and 21.3% of the phenotypic variation from the contributing parent Babax [28].

Despite numerous studies identifying QTLs and candidate genes on chromosome 4A, fine mapping of these regions for drought tolerance remains limited. This is due to diverse genetic backgrounds across genomic regions [29], large genomic intervals, and complexity of traits [10]. This underscores the importance of further candidate gene identification, marker development and understanding mechanisms for drought tolerance to develop drought-tolerant wheat. In this research, we evaluated four pairs of NILs with targeted yield QTL *qDT.4A.1* on chromosome 4A to identify candidate genes and SNP markers associated with drought tolerance traits via genotype–phenotype association analysis and identify mechanisms and functions of these candidate genes through in silico approaches. These findings will contribute to a better understanding of the genetic mechanisms underlying drought tolerance, supporting future wheat breeding efforts.

## 2. Results

### 2.1. Performance of NIL Pairs Under Well-Watered and Drought Conditions

**Figure 1:**
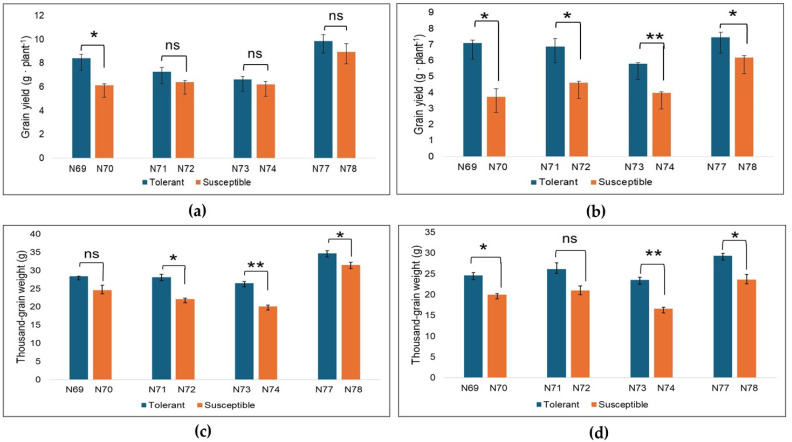
Grain yield (g·plant^−1^) of near-isogenic lines under well-watered (**a**), and drought-stressed conditions (**b**) and thousand-grain weight (g) of near-isogenic lines under well-watered (**c**), and drought-stressed conditions (**d**). ns = not significant at *p* ≤ 0.05; * = significant at *p* ≤ 0.05; ** = significant at *p* ≤ 0.01. Statistical analysis was performed via a *t*-test.

The grain yield (g·plant^−1^) and thousand-grain weight (g) of tolerant and susceptible isolines under control/well-watered and drought conditions are presented in Figure 1. Significant phenotypic variation in grain yield was observed between isolines from each NIL pair under well-watered and drought-stressed conditions. The NIL pairs N69 and N70 showed significant variations in grain yield under well-watered conditions, while no significant differences were observed in NIL pairs N71 and N72, N73 and N74, and N77 and N78 (Figure 1a). Under drought stress, significant differences in grain yield were observed for NIL pairs N69 and N70, N71 and N72, N77 and N78 (*p* ≤ 0.05), and N73 and N74 (*p* ≤ 0.01) (Figure 1b). The thousand-grain weight for the NIL pairs N69 and N70 did not significantly differ under well-watered conditions (Figure 1c), but differences were observed under drought stress (*p* ≤ 0.05). NIL pairs N71 and N72 presented the opposite results, whereas the NIL pairs N73 and N74 (*p* ≤ 0.01) and N77 and N78 (*p* ≤ 0.05) significantly differed under both conditions (Figure 1d). Among the NIL pairs, N77 and N78 exhibited the highest grain yield and TGW under all conditions.

Statistical analysis indicated by yield was significantly influenced by both genotypes (F = 16.07, *p* = 0.026, $\eta_{p}^{2}$ = 0.848) and environment (F = 32.8, *p* = 0.011, $\eta_{p}^{2}$ = 0.916) (Table S2). Importantly, a significant genotype × environment interaction for yield (F = 19.0, *p* = 0.022, $\eta_{p}^{2}$ = 0.864) was detected. In contrast, the genotype × environment interaction for TGW was not significant (*p* = 0.33) and exhibited a lower effect size $(\eta_{p}^{2}$ = 0.309) compared to yield (Table S2). Simple effects analysis revealed significant genotype effects on yield under control conditions explaining 39.3% variance (F = 20.684, *p* = 0.001, $\eta_{p}^{2}$ = 0.393), which increased substantially under drought stress to 71.3% (F = 79.6, *p* = 0.001, $\eta_{p}^{2}$ = 0.713) (Table S2). Pairwise comparisons showed a significant mean yield difference of 1.112 (*p* < 0.001) between tolerant and susceptible isolines under control conditions, which increased to 2.181 (*p* = 0.001) under drought conditions. In both environments, the tolerant isolines (carrying the target QTL) consistently outperformed susceptible isolines (Table S3).

### 2.2. SNP Markers in Targeted QTL Region

Among the 81,587 SNPs in the 90K SNP array, 31,821 SNPs were retained satisfying the selection criteria, and from this, a total of 8274 SNPs were assigned on chromosome 4A to identify markers with genotype–phenotype associations. Of these, six SNP markers on chromosome 4A showed consistent matching phenotype in NIL pairs. The SNP marker Kukri_rep_c109932_427 (622 Mb) showed a 100% genotype–phenotype match differentiating the tolerant and susceptible isolines in all NIL pairs (N69/N70, N71/N72, N73/N74, and N77/N78) (Table 1). Another five SNP markers Kukri_c27037_112 (529 Mb), Excalibur_rep_c80895_175 (531 Mb), Ex_c23792_486 (541 Mb), IACX2890 (605 Mb), and RAC875_c21369_425 (606 Mb) showed consistent associations in at least two NIL pairs (Table 1). In this study, we selected nearest SNP marker Kukri_c27037_112 positioned in the interval 529,988,237–529,988,318 to the target yield QTL *qDT.4A.1* (515,847,826–515,847,955) on chromosome 4A for further analysis.

### 2.3. Identified Candidate Genes

**Figure 2:**
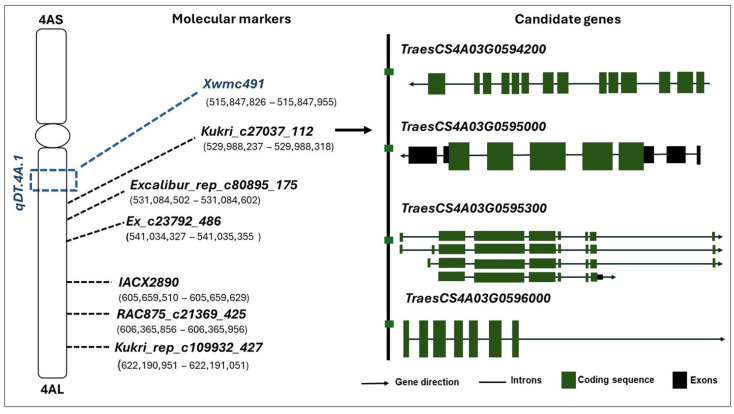
Identified single nucleotide polymorphism (SNP) markers linked with candidate genes near the target yield QTL *qDT.4A.1* on wheat chromosome 4A. Blue dashed line is the region of the target QTL *qDT.4A.1*. Structural features of candidate genes and SNPs obtained from wheat Chinese Spring genome versions 2.1.

We identified 40 putative candidate genes within 1 M bp in both upstream and downstream directions of SNP marker Kukri_c27037_112 linked to the yield QTL on chromosome 4A (Table S1). Four potential putative candidate genes were selected associated with drought tolerance-related traits (Figure 2 and Table 2). Candidate gene *TraesCS4A03G0594200* positioned in 529,233,709–529,233,891 interval encodes ring-finger E3 ubiquitin ligase involved in ubiquitin-protein transferase activity, and binding of protein, zinc ion, nucleotide, and metal ion (Figure 2 and Table 2). Candidate gene *TraesCS4A03G0595000* (529,987,389–529,989,560) encodes cysteine-rich receptor-like protein kinase and receptor kinase with functions involving protein kinase activity, protein serine/threonine kinase activity, and adenosine triphosphate (ATP) binding. Protein kinase family protein encoding gene *TraesCS4A03G0595300* (530,127,677–530,127,712) is involved in protein kinase activity and binding of ATP and adenosine diphosphate (ADP). Another candidate gene *TraesCS4A03G0596000* (530,297,158–530,297,257) encodes receptor kinase 1 with functions in protein kinase activity and ATP binding (Figure 2 and Table 2).

Functional enrichment analysis based on KEGG pathway [30,31] retrieved in STRING database showed that these candidate genes were involved in different processes and pathways. We identified important KEGG pathways associated with drought response; protein processing in endoplasmic reticulum (ER), and mitogen-activated protein kinase (MAPK) signaling pathway (Figure S1).

### 2.4. In Silico Gene Expression Analysis

**Figure 3:**
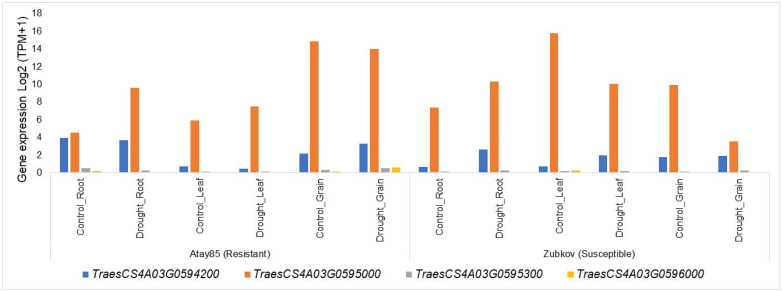
In silico gene expression of selected candidate genes in root, leaf, and grain tissues of Atay85 (resistant) and Zubkov (susceptible) varieties based on WheatOmics.

The gene expression data of six selected putative candidate genes across root, leaf, and grain tissues under control/well-watered and drought condition using Atay85 (resistant) and Zubkov (susceptible) varieties are presented in Figure 3. Candidate gene *TraesCS4A03G0594200* was more highly expressed in the grain tissues of resistant Atay85 variety under drought stress than those of the control (Figure 3). Candidate gene *TraesCS4A03G0595000* showed increased expression in both root and leaf tissues under drought compared to the control, while maintaining relatively stable expression in grain tissue with 14.8 transcript per million (TPM) in the control and only a slight reduction to 14.0 TPM under drought stress. Both *TraesCS4A03G0595300* and *TraesCS4A03G0596000* demonstrated increased expression in the grains of the resistant variety under drought stress compared to the control (Figure 3). The percentage relative differences in gene expression between resistant and susceptible varieties are shown in Figure S2. In root tissues under control conditions, relative differences were observed for *TraesCS4A03G0594200, TraesCS4A03G0595000, TraesCS4A03G0595300,* and *TraesCS4A03G0596000*; however, these differences decreased under drought conditions. In leaf tissue, negative relative differences were observed under both control and drought conditions. In contrast, grain tissues showed notable relative differences under control conditions, with even greater differences observed under drought stress. Among the candidate genes, *TraesCS4A03G0595000* and *TraesCS4A03G0596000* exhibited the highest relative differences under drought conditions (Figure S2).

## 3. Discussion

Drought significantly impacts crop yield, particularly during critical growth stages in wheat. Identifying markers and genes associated with drought tolerance is paramount for expediting the development of drought-tolerant wheat varieties. This study characterized four NIL pairs targeting the yield QTL on chromosome 4A under well-watered and drought-stressed conditions. Drought stress caused significant variation in grain yield and thousand-grain weight across NIL pairs. Similar observations were reported by [32] in their transcriptomic studies with seven-day drought, with yield reductions of 35% (tolerant) and 44% (susceptible lines). Ref. [33] observed 13% and 26% reductions in total grain weight for tolerant and susceptible cultivars, respectively. Ref. [34] also observed reductions in thousand-grain weight and grain yield after seven days of drought in hybrid crosses and parents. Although these data demonstrate the impact of a 7-day drought, it is not possible to distinguish between physiological tolerance and drought avoidance mechanisms. Future studies should include leaf water potential, stomatal conductance, and osmotic adjustment to better understand the mechanisms underlying drought tolerance.

Based on statistical results, the developed NILs in this study demonstrated stress-specific adaptation. The significant genotype × environment interaction indicates that the yield performance of NILs was differentially influenced by environmental conditions (drought vs. control) providing insights into tolerance mechanisms associated with the target QTL. The non-significant genotype × environment interaction for TGW may imply that this trait is relatively stable across control and drought conditions in this study. The main independent effects of genotype (*p* = 0.003) and environment (*p* = 0.33) contributed significantly to variation in TGW rather than their interaction. Similarly, Ref. [35] reported a significant genotype effect on TGW and a strong influence of water availability on grain yield. The presence of the QTL in their QYld.idw-3B++ lines resulted in higher grain weight and increased grain number [35]. The simple effects analysis in this study further revealed that the increase in effect size from 0.393 under control to 0.713 under drought conditions indicates an amplified genotypic effect under stress suggesting that tolerant isolines (with the target QTL) exhibited enhanced stress-responsive performance under drought conditions. Similarly, Ref. [36] reported a large QTL effect on rice yield under drought stress; the QTL *qDTY12.1* contributed to increased biomass, harvest index and plant height. Furthermore, the pairwise comparisons confirm the consistent yield advantage of tolerant isolines under drought, thereby reducing the yield gap. This validates the advantage of tolerant isolines carrying the target QTL over susceptible isolines lacking it. The use of NILs in this study enables precise evaluation of the specific contribution of the target QTL to drought tolerance.

Across the NIL pairs, tolerant isolines carrying the target QTL consistently exhibited higher grain yield and TGW, as compared to isolines lacking the QTL under both control/well-watered and drought conditions. This superior performance suggests that tolerant isolines maintain yield under drought, possibly through more effective carbohydrate translocation to the grains, whereas susceptible isolines may experience reduced grain filling under stress. Similar observations have been reported by [37] who noted that drought-tolerant wheat is more yield-stable than drought-sensitive varieties, particularly under variable climate conditions. The observed variation in grain yield and TGW among NILs underscores their value as breeding material for marker and candidate gene identification. Our findings are consistent with previous studies on the same genotypes [14,15] and with reports showing greater genetic variation under drought stress than under well-watered conditions [38,39]. These results reinforce the idea that drought acts as a selective pressure, shaping genetic variation and influencing wheat grain yield.

The targeted QTL in this study accounts for 27% of the yield variation under drought stress [20] highlighting its major contribution to drought-adaptive yield performance. Six SNP markers consistently showed genotype–phenotype associations in at least two NIL pairs targeting the yield-related QTL on chromosome 4A, highlighting the importance of this genomic region. The low SNP consistency has been reported in some studies using NILs due to complexity of quantitative traits and background specificity. For example, Ref. [16] identified five SNP markers with consistent contrasting genotypes of NILs within the 7AL QTL region for heat tolerance in wheat. Ten out of 4334 SNPs were identified with consistent genotype–phenotype associations between tolerant and susceptible isolines for pre-harvest sprouting [16], and only six SNPs were identified between NIL pairs for drought tolerance in wheat [40]. Genotyping was performed using a high-density 90K SNP array, enabling wide coverage for detection of genetic variation. The subsequent genotype-phenotype association analysis has proven effective in identifying candidate genes and markers, such as those associated with plant height [15] and root traits, including UDP-glycosyltransferase (UGT) and leucine-rich repeat receptor-like protein kinase (LRR-RLK) [14]. Other studies have identified several meta-QTLs with functions related to GY under drought, heat, and combined drought and heat stress [28]. The SNP marker Kukri_c27037_112 was not only the closest marker to the target QTL but also demonstrated 100% genotype–phenotype association in the NIL pairs. Similar studies suggest that SNPs showing perfect genotype–phenotype associations in at least two NIL pairs have been successfully used to identify markers linked to important traits such as plant height [15], root traits [14], and heat tolerance [16]. Although this SNP marker is 14.1 Mb away from the target QTL, it may still be associated with the target locus and serve as an informative marker and initial resource for future validation studies. In another study, an SNP marker located 10.1–26.2 Mb from target QTL showed 81.8% accuracy in KASP conversion for drought tolerance [40]. Meta-QTL analysis revealed that flanking markers Kukri_c27037_112/barc340a are associated with grain yield, grain weight, grain number, thermotolerance, and plant height [41]. Additional validation across different backgrounds and populations is required to confirm the utility of these SNP markers.

Several studies have reported that the 4AL region contains significant QTL hotspots for drought tolerance QTL. For example, the QTL *QTKW.ndsu.4A.2* for thousand kernel weight under drought was found on chromosome 4AL [42], with similar genomic position in the grain yield QTL associated with grain yield-related traits under drought stress found in the chromosome 4AL [22]. Numerous studies have reported QTLs for yield-related traits on chromosome 4A, including grain number and biomass per plant [15], root characteristics [14], and spike and grain traits [43]. The QTL *qDTGW-4A* on chromosome 4A explained 3.26% of the phenotypic variation under drought, and two QTLs (*qSTI-4A.1* and *qSTI-4A.2*) for the stress tolerance index accounted for 3.37–4.22% of the phenotypic variation [44]. SSRs linked to grain yield (GY) and the stress susceptibility index were also found on chromosome 4A, explaining 14–41% of the variation across growing seasons [22]. QTL associated with drought tolerance indices identified via SNP markers and association mapping, indicated that the chromosome 4A region plays a major role in drought tolerance [24]. Additionally, the QTL QWSC-4A for water-soluble carbohydrates (WSC), which explains 10.47% of phenotypic variation, increased the WSC [45]. Further characterization of this QTL-rich region could identify significantly linked candidate genes for yield-related traits, particularly drought tolerance.

Linked to the identified SNP marker Kukri_c27037_112, four putative candidate genes associated with drought response were identified on chromosome 4A. Candidate gene *TraesCS4A03G0594200* encodes RING-finger E3 ubiquitin ligase, which is an important drought response regulator through posttranslational activity, specifically the ubiquitination and degradation of regulatory proteins [46]. For example, E3 ubiquitin ligase activity of drought hypersensitive (DHS) gene in rice (*Oryza sativa* L.) resulted in the reduction in drought tolerance and downregulation of wax biosynthesis [47]. Some studies reported the function of RING finger E3 ubiquitin ligase genes such as TaAIRP2-1B in controlling spike length [48], and TaSDIR1-4A in contributing grain size [49]. RING-finger E3 ubiquitin ligases play a critical role in regulating yield and thousand-grain weight under drought stress conditions. For example, the interaction and subsequent degradation of TaAGPS mediated by the RING-finger E3 ligase TaGW2-6A-CS influences AGPase activity and starch granule formation, thereby increasing grain size [50]. In rice, the E3 ubiquitin ligase *OsRGLG6* ubiquitinates the deubiquinating enzyme *OsOTUB1* promoting degradation and resulting in the co-regulation of multiple genes involved in hormone signaling, nitrogen utilization and responses to biotic and abiotic stresses. This coordinated regulation between *OsRGLG6* and *OsOTUB1* contributes to enhanced yield and improved drought tolerance [51]. Similarly, overexpression of the U-box gene *TaPUB1* in wheat enhances drought tolerance by maintaining higher leaf water status and increasing antioxidant capacity [52]. A U-box E3 ubiquitin ligase OsPUB67 positively controls drought tolerance in rice through improving reactive oxygen scavenging ability, stomatal closure, and gene expression regulation associated with abiotic response in rice [53]. In wheat, *TaGW2,* a finger E3 ligase gene, positively controls drought resistance and leads to higher yield through degradation of TaARR12 demonstrating the significant role of *TaGW2* in mediating the trade-off between grain yield and drought tolerance [46]. In another study, TaSDIR1-4A facilitates the polyubiquitination and degradation of TaWRKY29, resulting in its activation and increased expression and consequently fine tunes the regulation of abscisic acid (ABA) signaling and enhancing drought tolerance in wheat [54]. Based on their in silico transcriptomic data analysis, *TaGW2* is highly expressed in kernels. Similarly, in this study, the increased variation in *TraesCS4A03G0594200* encoding ring finger ubiquitin ligase at drought condition, compared with the control, may suggest its ability to manage the stability of proteins and nutrient translocation during stress condition.

Another candidate gene, *TraesCS4A03G0595300* encodes protein kinase family protein with functions in protein kinase activity and ATP binding. Lastly, two candidate genes, *TraesCS4A03G0595000* and *TraesCS4A03G0596000,* encode receptor kinases that play an important role in plant development and responses to drought, salt and cold stresses [55]. It has been reported that protein kinase families are involved in plant development and abiotic responses. For example, the overexpression of wheat sucrose non-fermenting-1-related protein kinases (SnRKs) *TaSnRK3.23B* confers drought tolerance in Arabidopsis (*Arabidopsis thaliana* L.) through ROS scavenging [56]. The receptor protein kinase and serine/threonine protein kinase-encoding gene helps plant growth and development under abiotic stress [57]. A wheat SNF1-type serine/threonine protein kinase (*TaSnRK2.4*) gene significantly enhanced drought, salt, and cold tolerance by acting as a multifunctional regulatory factor in Arabidopsis, regulating growth and osmotic potential under normal and stress conditions [58]. Using transcriptomic analysis in Arabidopsis, an autophagy-dependent protein kinase ATG1 was found associated with drought tolerance through ABA synthesis regulation and has an important role in signaling network in response to stress [59]. The expression patterns of wheat leucine-rich repeat receptor-like protein kinase (LRR-RLK) suggest that this gene may be related to stress responses during drought [60]. The gene encoding serine/threonine protein kinase LRK10-like (*TraesCS3D02G011300*) was found to regulate grain weight [61] indicating its important role in grain yield-related traits. Protein kinases play essential roles in plant development, stress sensing and signal transduction under various stress conditions [62]. For instance, the protein kinase TaSnRK2.4 has been shown to regulate thousand-grain weight, and its interaction with TaLTP3 enhances abiotic stress tolerance [63]. Another study demonstrated that the interaction between the protein kinases OsMAPK5–OsWRKY72 mediates to auxin-modulated responses and regulates grain length and weight in rice [64,65], and reported that receptor-like kinase LecRK-VIII.2, an upstream component of the MAPK signaling pathway, regulates seed yield in Arabidopsis by coordinating silique number and seed size. This kinase is also involved in regulating cell expansion, thereby influencing seed growth. In the present study, the higher variation in expression of the receptor kinase-encoding putative candidate genes *TraesCS4A03G0595000* and *TraesCS4A03G0596000* in grain tissue under drought conditions, compared with the control, may suggest their involvement in stress perception and grain development under drought. Collectively, these putative candidate genes may function coordinately under drought conditions, whereby *TraesCS4A03G0595000* and *TraesCS4A03G0596000* (encoding receptor kinases) may act as stress sensors and initiate signaling cascades, which are subsequently transmitted to *TraesCS4A03G0595300* (encoding a protein kinase). In turn, *TraesCS4A03G0594200* (encoding a RING-finger ubiquitin ligase) may regulate downstream responses during grain development, potentially contributing to yield stability under drought stress. Although we conducted in silico gene expression analyses of selected candidate genes using the WheatOmics database, comparative expression analyses in the Babax/Dharwar Dry background are required to fully elucidate the molecular mechanisms underlying drought tolerance. The important roles and potential interactions of these putative candidate genes necessitate further confirmation through transcriptomic studies, fine mapping, and functional gene validation to substantiate these preliminary findings.

KEGG pathway analysis [30,31] and protein–protein interaction network prediction data may be useful in predicting gene function and relationships with other genes [66] thus contributing to a better understanding in their stress adaptation and enhancing drought tolerance in wheat. The protein–protein interaction functional enrichment analysis in STRING database revealed that the selected candidate genes were involved in two significant KEGG pathways [30,31]: protein processing in endoplasmic reticulum (ER), and MAPK signaling pathway. The production of one-third of the cellular proteins associated in signaling and cell response usually happens in the ER and components of protein processing in this organelle have been associated with abiotic stress response [67]. Abiotic stresses such as drought, heat, and salinity cause stress in the ER [68,69,70]. Several strategies such as unfolded protein response (UPR), ER-associated degradation (ERAD), and autophagy have been identified to reduce stress in ER [71], making these potential targets for genetic engineering to improve stress tolerance in plants [72]. Examples include the overexpression of BiP, one of the most abundant chaperone proteins in the ER lumen, which confers high drought tolerance with unknown mechanisms associated with ER functioning in soybean (*Glycine max* (L.) Merr.) [73]. In wheat, the BiP expression increases when cells experienced osmotic stress-induced cell death caused by an apoptosis inhibitor [74]. In addition, the involvement of the *Gh_D09G2402.1 (GhCNX)* gene in the ER protein processing pathway was reported, specifically the *GhCNX6* gene, which is responsible for enhancing drought tolerance in cotton (*Gossypium hirsutum* L.) [75]. The study by [76] reported that protein processing in the ER pathway was significantly enriched in the drought-tolerant line YE8112. This tolerance was associated with increased accumulated heat shock proteins and seed storage proteins, as well as enhanced post-translational modifications, contributing to improved kernel filling under drought conditions in maize (*Zea mays* L.) kernel [76]. In Arabidopsis, Atp24δ8 enhances plant adaptation during stress through regulating unfolded protein response (UPR) gene expression and maintaining ER homeostasis [77]. Furthermore, the localization of the abiotic stress-responsive gene *TaUSP* in ER suggests its involvement in the UPR pathway, where it enhances root morphology and thereby contributes to improved drought tolerance [78].

The candidate genes identified in this study were also involved in the mitogen-activated protein kinase (MAPK) signaling pathway, which plays an important role in response to abiotic stresses [79] by transmitting signals receive from the exterior part of the cell [80]. The overexpression of GhMAPK16 associated with signal transduction pathway enhances the drought tolerance in Arabidopsis [81]. Previous studies reported examples of MAPK involved in drought tolerance, such as GhMKK3 in cotton [82], GhMAPK3 in Arabidopsis [83] and SlMPK3 in tomato (*Solanum lycopersicum* L.) [84]. In wheat, MAPKs such as TaMPK6 and TaMPK16 mediate plant responses in drought, high salinity, phosphorus and nitrogen deprivation through MAPK cascade modules [85]. Increased expression levels of MAPK3 and MAPK6 genes, including proline content in the most tolerant genotypes under severe drought stress conditions, were observed to be associated with the role of MAPK in enhancing drought tolerance in wheat [86]. In a recent study, the interaction between ABA and MAPK signaling pathways—which includes the signaling modules of cysteine-rich receptor-like kinases (CRKs) activating the protein kinase (MAPK) cascade and phosphorylating abscisic acid-responsive transcription factors—led to enhanced drought resistance in rice [87]. Studies in transcriptomic profiling revealed that MAPK signaling is one of the essential biological pathways in drought response [88]. MAPK signaling is also involved in controlling plant growth and development [89,90]. The upregulated expression of MAPK3 in wheat glumes has been identified as part of the plant stress response during drought at the early grain-filling stage [91]. In another study, the OsMKKK10-OsMKK4-OsMAPK6 cascade was shown to regulate grain size and weight through modulation of cell proliferation highlighting the critical role of the MAPK signaling pathway in improving grain-related traits in rice [92]. Additionally, overexpression of the novel gene *OstMAPKKK5*, which encodes a truncated protein lacking the kinase domain, was identified as a positive regulator of plant height, grain yield and yield-related traits (including grain width grain length, thousand-grain weight, and grain number per panicle) in rice. This enhancement was attributed to increased accumulation of endogenous gibberellins (GA1, GA3 and GA4) and promotion of cell enlargement [93]. Collectively, these studies demonstrate that the MAPK signaling pathway participates in multiple biological processes in plants, including stress signaling, hormone regulation, cell proliferation, and grain development, thereby contributing to yield stability under stress conditions. The identification of molecular markers and candidate genes associated with the MAPK pathway through functional studies, marker-assisted selection, and genome-assisted breeding is essential in crop improvement breeding program [94].

In summary, we identified SNP markers and candidate genes through the integration of genotype–phenotype association analysis using 90K SNP array and evaluation of NILs targeting the yield QTL (*qDT.4A.1*) on chromosome 4A. The notable relative differences in gene expression in grain tissue as compared to leaf and roots under drought versus control conditions depicts their important function in grain development under drought stress conditions. The in silico gene expression of selected candidate genes provides an initial foundation for understanding NIL responses under drought stress. While upregulation of putative candidate genes associated with drought response was identified, this alone is insufficient as functional proof. Future studies should include transcriptomics, qRT-PCR validation, proteomics, metabolomics, fine mapping, or functional studies (e.g., CRISPR/Cas9 gene editing) to validate the role of these genes in drought response. While we successfully identified SNPs and candidate genes using NILs in this study, several limitations should be considered. First, NIL development is time consuming, but with the embryo-based fast cycling technique [10] used in this study we have accelerated NIL development. The ability to examine genetic interactions is limited due to single introgression segments in NILs; therefore, QTL identification is background-dependent-specific [95]. The identified SNPs remain putative, as their designation is based on functional annotation and physical proximity to the associated SNP marker. To ensure reliability of the identified SNP markers in this study, further validation across diverse mapping populations and environmental conditions is required before these SNPs can be converted into breeder-friendly KASP assays. Moreover, the identified candidate genes remain putative, as their designation is based on functional annotation and physical proximity to the associated SNP marker. Functional validation such as transgenic approaches or gene-editing techniques was not performed in this study to confirm the causal roles of the associated SNPs and candidate genes. Additionally, expression analyses of the candidate genes were not conducted, which limits our understanding of their potential mechanisms and functional roles in drought tolerance. These limitations highlight the importance of validating the results of this study through comprehensive functional investigations including transcriptomics and qRT-PCR validation, proteomics, metabolomics, and fine mapping. Such approaches are essential to confirm gene-trait associations under drought conditions and to establish their practical utility in breeding programs.

## 4. Materials and Methods

### 4.1. Development of NIL Population Using Heterogenous Inbred Family Method

This study used four pairs of NILs derived from a cross between the wheat varieties Babax and Dharwar Dry. Babax is a semidwarf variety known for its broad adaptability and high drought tolerance [96], whereas Dharwar Dry comes from Central India where wheat cultivation primarily relies on residual moisture [22]. These NILs were developed specifically to target yield-related QTL (*qDT.4A.1*) associated with drought tolerance on chromosome 4A. NILs were developed through fast generation cycling system (FGCS) based on immature embryo culture [10,97] in combination with the heterogeneous inbred family (HIF) [98] following the described protocol of [10]. Briefly, heterozygous plants were selected from F2 to F4 generation using the single seed descent method. Selection of heterozygous plants was performed using the linked marker Xwmc491 targeting the yield QTL (*qDT.4A.1*) on chromosome 4A [20]. From F4 to F7 generations, six to eight plants from each heterozygous progeny were genotyped using the linked marker to select one heterozygous plant for selfing and advancement. In F7, two isolines homozygous at the target locus but contrasting in parental alleles were selected. F7 plants with >98% recipient genome recovery was advanced to F8 (99%) constituting the putative NILs used for subsequent phenotyping and genotyping. The observed differences in drought tolerance make these progenies ideal for studying drought-related QTLs. These NIL pairs exhibited significant differences in biomass, yield [15] and root traits [14].

### 4.2. Generation Advancement Through Embryo Culture Based Fast Generation Cycling System

The embryo culture-based fast generation cycling system (FGCS) was used for the F3 to F7 generation advancement following the protocol described by [10,97]. In short, immature embryos were collected from developing grains approximately 12–14 days after anthesis and cultured under sterile conditions in Petri dishes. The cultured embryos were incubated in a dark plant growth chamber to promote germination. Once germinated, seedlings were transferred to a growth room maintained at 22 °C with a 16 h fluorescent lighting to induce rooting. Young seedlings with root lengths of at least 2.0 cm were transferred into plastic trays containing growth media and maintained in plant growth chambers until grains were ready for subsequent cycles of embryo culture and generation advancement.

### 4.3. Experimental Design and Treatments

The NIL evaluation was conducted in a temperature-controlled glasshouse facility at The University of Western Australia (UWA) in Perth, Western Australia. The average temperature was 19 °C (ranging from 9.9 to 40.7 °C), with relative humidity of 67%. Seeds were sown in cylindrical PVC pots (9 cm × 50 cm) filled with 2.5 kg of air-dried soil. The experiment had a randomized complete block design with two separate treatments, each with three replications. The treatments were as follows: (1) well-watered (control), with pots watered regularly to 80 to 100% pot capacity until maturity; (2) drought stress, applied at the onset of anthesis and lasted for 7 days, followed by rewatering until maturity. Drought stress was imposed continuously for seven days during the sensitive anthesis stage. Timing and duration were based on previous studies [10,32] to induce a measurable drought response without causing severe damage. Soil moisture was monitored to ensure consistent stress, and plants were re-watered after seven days to allow recovery until maturity. The grain yield (g·plant^−1^) was measured as the total grain weight per plant, and the thousand-grain weight (TGW) was determined by counting and weighing 100 grains per entry with three replications and converted to 1000 grain weight. A paired two-sample *t*-test at significance levels of 0.05 was done to compare the differences between the isolines in each NIL pair.

A General Linear Model (GLM) analysis using SPSS v29.0 was conducted to evaluate drought tolerance of NILs based on differences in yield and TGW. Genotype (tolerant vs. susceptible) and environment (drought vs. control) were treated as fixed factors, while NIL pairs and all interaction terms were included in the model to appropriately partition variance components. Statistical significance was defined at *p* < 0.05. Pairwise mean comparison was analyzed using Bonferroni correction, and effect sizes were reported as partial eta squared $(\eta_{p}^{2}$), representing the proportion of variance explained by each factor interaction.

### 4.4. SNP Genotyping and Marker Identification

The NIL pairs were genotyped using a high-density 90K Illumina SNP array comprising 81,587 SNPs [99]. SNP clustering and calling were performed with GenomeStudio 2.0 software [100]. The raw SNP data were filtered to exclude SNPs with a call frequency lower than 0.8, more than 20% missing data points, monomorphic markers, markers with a minor allele frequency less than 0.05, and heterozygous calls exceeding 0.25. The genotypic data was filtered based on chromosomal position [101,102]. Using the corresponding phenotype data, the filtered SNPs were divided and assigned into two classes: tolerant and susceptible. Notably, the SNP markers especially adjacent to Xgwm397 to Xwmc491 on chromosome 4A, which showed consistent genotype–phenotype associations in at least two NIL pairs, were selected for further analysis.

### 4.5. Candidate Gene Identification, Annotation, and In Silico Analysis

The TriticeaToolbox (T3) database (https://wheat.triticeaetoolbox.org/, accessed on 10 June 2025) was used to retrieve sequences of the selected SNP markers, which were aligned with the wheat reference genomes RefSeq v2.1 [101,102] via BLASTN tools in GrainGenes database (https://graingenes.org/ (accessed on 10 June 2025). The IWGSC Chinese Spring genome database (https://urgi.versailles.inra.fr/jbrowseiwgsc/gmod_jbrowse/ (accessed on 10 June 2025)) was used to identify genes related to yield traits on chromosome 4A. Descriptions of putative candidate genes were derived from GrainGenes (https://graingenes.org/GG3/ (accessed on 10 June 2025)), EnsemblPlants (http://plants.ensembl.org/, accessed on 10 June 2025), NCBI (https://www.ncbi.nlm.nih.gov/ (accessed on 10 June 2025), WheatOmics 1.0 (http://wheatomics.sdau.edu.cn/ (accessed on 10 June 2025), and IWGSC Annotation v2.1.

In silico gene expression analysis of putative candidate genes was done using the publicly available wheat expression database Triticeae Multi-omics center (http://wheatomics.sdau.edu.cn/ (accessed on 10 June 2025) [103]. Gene expression data expressed as transcripts per million (TPM) values in root, leaf, and grain tissues under normal and drought conditions were analyzed using Atay85 as a resistant variety and Zubkov as a susceptible variety, as per the Hexaploid Wheat Expression Database WheatOmics v1.0 (http://wheatomics.sdau.edu.cn/ (accessed on 10 June 2025) [103]. Normalized TPM values for each tissue and condition were used for descriptive comparison and visualization. Using TPM values retrieved from WheatOmics, relative difference between Atay85 (resistant) and Zubkov (susceptible) were calculated as [104]:

$$Relative\;difference\;(\%)=\frac{A-B\;}{(A+B)/2}\times \;100$$

where A and B are TPM values under control or drought. Percent differences illustrate variation between tolerant and susceptible lines.

**Figure 4:**
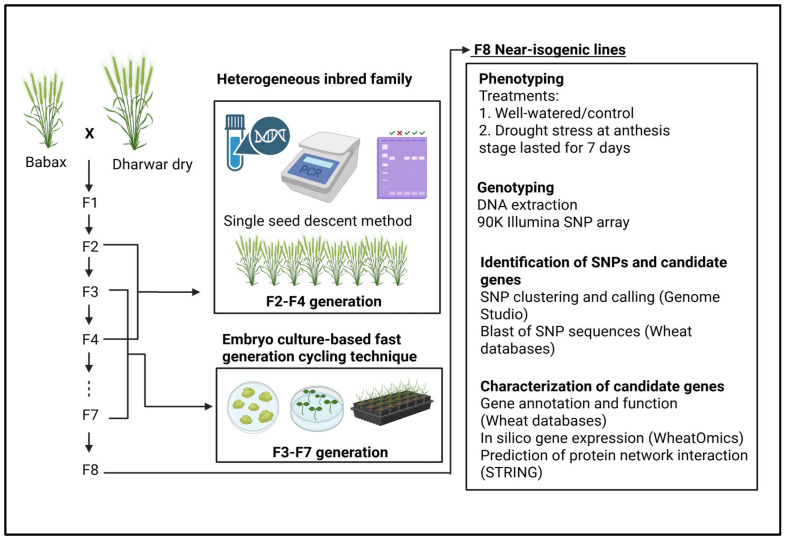
Summary of near-isogenic line development and evaluation for marker and candidate gene identification using genotype–phenotype association and in silico analysis. Figure created in BioRender. Caguiat, J. (2026) https://BioRender.com/qmkxum1 (accessed on 11 March 2026).

The protein–protein interaction networks were predicted using STRING database (https://string-db.org/ accessed on 4 November 2025) by entering the protein sequences of candidate genes in the search bar with default settings, and those with >40% identity were included in the network interaction analysis [105], aiming to explore the indirect influence of genes on traits by examining their interactions within networks. To visualize the gene expression, heatmap and results generated from STRING database, data was plotted in an online platform https://www.bioinformatics.com.cn (accessed on 4 November 2025) [106]. A methodological overview is presented in Figure 4.

## 5. Conclusions

We developed near-isogenic lines targeting the yield QTL (*qDT.4A.1*) on chromosome 4A using a combined embryo culture-based fast generation cycling system and the heterogenous inbred family method. Yield and TGW were evaluated under well-watered and drought conditions. Tolerant isolines containing the positive QTL consistently outperformed susceptible isolines. The reduced TGW in susceptible isolines may reflect limited nutrient translocation under drought conditions.

Genotype × environment interactions strongly influenced the yield performance of NILs in this study, confirming that drought response involves environmentally dependent genetic effects. In contrast, TGW appears comparatively stable, as it does not significantly depend on these interactions. The consistent and amplified yield advantage of tolerant isolines carrying the target QTL under drought, together with large effect sizes and significant interaction terms, provides statistically robust evidence for stress-adaptive yield responses associated with the QTL.

Using genotype–phenotype association analysis, we identified SNP marker Kukri_c27037_112 tightly linked with four candidate genes involved in drought response. Protein–protein interaction enrichment analysis suggested these genes participate in protein processing in the endoplasmic reticulum and MAPK signaling pathways. In silico gene expression analysis indicated upregulation of these candidate genes near the SNP marker, but further transcriptomic, proteomic, and functional validation studies are needed to confirm these findings. The identified SNPs represent promising candidates for future KASP assay development pending experimental validation. Functional studies and transcriptomic analyses are necessary to confirm and validate the role of these candidate genes under drought stress. Overall, this study demonstrated the use of NILs in SNP and candidate gene identification, providing valuable insights into drought tolerance mechanisms and laying the groundwork for future research on drought tolerance in wheat.

## Figures and Tables

**Table 1:** Single nucleotide polymorphism (SNP) genotype profile matches the phenotypic differences between resistant and susceptible isolines of near-isogenic line (NIL) pairs.

SNP Markers	NIL1		NIL2		NIL3		NIL4	
	N69 +	N70 −	N71 +	N72 −	N73 +	N74 −	N77 +	N78 −
Kukri_c27037_112	BB	BB	BB	AA	NC	NC	BB	AA
Excalibur_rep_c80895_175	BB	BB	BB	AA	NC	NC	BB	AA
Ex_c23792_486	BB	BB	BB	AA	BB	BB	BB	AA
IACX2890	AA	BB	BB	NC	AA	AA	AA	BB
RAC875_c21369_425	AA	BB	BB	NC	AA	AA	AA	BB
Kukri_rep_c109932_427	BB	AA	BB	AA	BB	AA	BB	AA

+ tolerant; − susceptible; NC, No Calls.

**Table 2:** Identified putative candidate genes with their physical position, description, and gene ontology derived from NCBI, Ensembl, and WheatOmics.

Candidate Gene	Start	End	Description and Gene Ontology
*TraesCS4A03G0594200*	529,233,709	529,233,891	Description: RING-finger E3 ubiquitin ligase, putative
			GO:0005515 MF: protein binding
			GO:0008270 MF: zinc ion binding
			GO:0004842 MF: ubiquitin-protein transferase activity
			GO:0000166 MF: nucleotide binding
			GO:0046872 MF: metal ion binding
			GO:0006511 BP: ubiquitin-dependent protein catabolic process GO:0019941 BP: modification-dependent protein catabolic process
			GO:0000209 BP: protein polyubiquitination
			GO:0007093 BP: mitotic cell cycle checkpoint
			GO:0016567 BP: protein ubiquitination
			GO:0051301 BP: cell division
*TraesCS4A03G0595000*	529,987,389	529,989,560	Description: Receptor kinase
			GO:0004672 MF: protein kinase activity
			GO:0004674 MF: protein serine/threonine kinase activity
			GO:0005524 MF: ATP binding
			GO:0005886 BP: plasma membrane
			GO:0016021 BP: integral component of membrane
			GO:0006468 BP: protein phosphorylation
			GO:0042742 BP: defense response to bacterium
*TraesCS4A03G0595300*	530,127,677	530,127,712	Description: Protein kinase family protein
			GO:0004672 MF: protein kinase activity
			GO:0005524 MF: ATP binding
			GO:0043531 MF: ADP binding
			GO:0006468 BP: protein phosphorylation
*TraesCS4A03G0596000*	530,297,158	530,297,257	Description: Receptor kinase
			GO:0004672 MF: protein kinase activity
			GO:0005524 MF: ATP binding
			GO:0006468 BP: protein phosphorylation

## Data Availability

All data presented or analyzed in the study are included in the article and Supplementary Material. Further inquiries can be directed at the corresponding authors.

## Supplementary Materials

The following supporting information can be downloaded at: https://www.mdpi.com/article/10.3390/plants15060921/s1, Table S1: Candidate genes within 1 M bp in both upstream and downstream directions of SNP marker Kukri_c27037_112 near the target QTL on chromosome 4A. Table S2. Tests of between-subjects for yield and thousand-grain weight (TGW) across genotypes (tolerant vs. susceptible) and environments (drought vs. control). Table S3. Pairwise comparisons of yield and thousand-grain weight (TGW) of near-isogenic lines (NILs) under drought and control condition. Figure S1: Significant enrichment generated from STRING database using protein–protein interaction analysis of selected candidate genes (a) *TraesCS4A03G0594200*, (b) *TraesCS4A03G0595000*, (c) *TraesCS4A03G0595300*, and (d) *TraesCS4A03G0596000*. BP, Biological process; MF, Molecular function; CC, Cellular component. Figure S2. Relative percentage differences in gene expression of selected putative candidate genes across root, leaf, and grain tissues of resistant and susceptible varieties under control/well-watered and drought conditions. Data was retrieved from WheatOmics database.

## Abbreviations

The following abbreviations are used in this manuscript:

ABA	Abscisic acid
ADP	Adenosine diphosphate
ATP	Adenosine triphosphate
bp	Base pair
BP	biological process
CC	Cellular component
CRK	Cysteine-rich receptor-like kinases
DHS	Drought hypersensitive
ER	Endoplasmic reticulum
ERAD	ER-associated degradation
FGCS	Fast generation cycling system
GY	Grain yield
HIF	Heterogeneous inbred family
KASP	Kompetitive Allele Specific PCR
LRR-RLK	Leucine-rich repeat receptor-like protein kinase
MAPK	Mitogen-activated protein kinase
Mb	Million base pairs
MF	Molecular function
NILs	Near-isogenic lines
QTL	Quantitative trait loci
TGW	Thousand-grain weight
TPM	Transcript per million
UGT	UDP-glycosyltransferase
UPR	Unfolded protein response
UWA	University of Western Australia
WSC	Water-soluble carbohydrates
