# Supplementary material for: Identification of SNPs and Candidate Genes Associated with Major Drought Tolerance QTL on Wheat Chromosome 4A
Source: Plants (Basel). 2026 Mar 16;15(6):921. doi: 10.3390/plants15060921 (PMC13029921; doi:10.3390/plants15060921)
Supplement: Supplementary file 1 [file plants-15-00921-s001.zip › Figure S1.pdf]

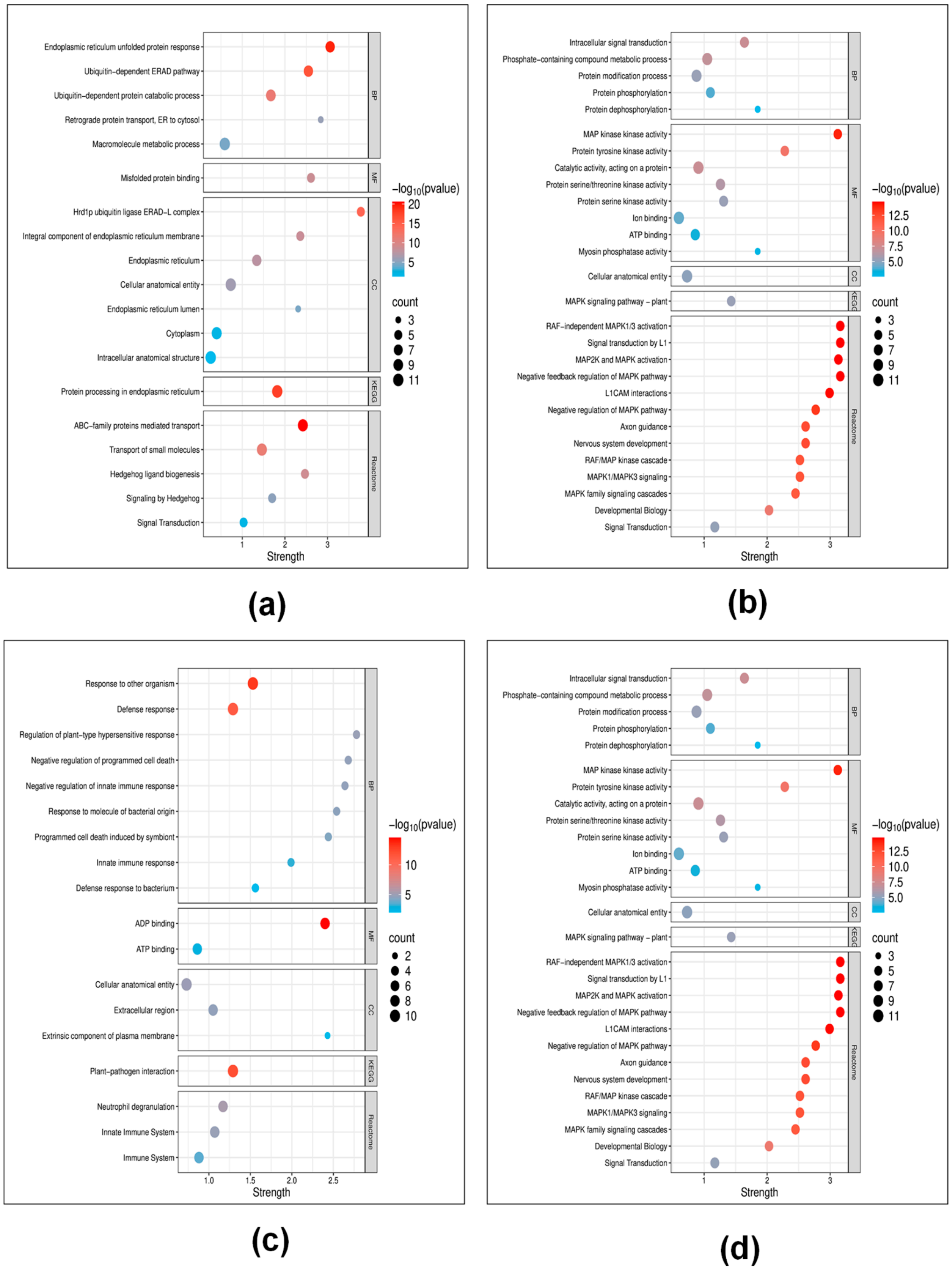

Figure S1. Significant enrichment generated from STRING database using protein-protein interaction analysis of selected candidate genes a) *TraesCS4A03G0594200*, b) *TraesCS4A03G0595000*, c) *TraesCS4A03G0595300*, and d) *TraesCS4A03G0596000*. BP, Biological process; MF, Molecular function; CC, Cellular component.

 https://doi.org/10.3390/plants15060921
