# Supplementary material for: Identification of SNPs and Candidate Genes Associated with Major Drought Tolerance QTL on Wheat Chromosome 4A
Source: Plants (Basel). 2026 Mar 16;15(6):921. doi: 10.3390/plants15060921 (PMC13029921; doi:10.3390/plants15060921)
Supplement: Supplementary file 1 [file plants-15-00921-s001.zip › Figure S2.pdf]

plants

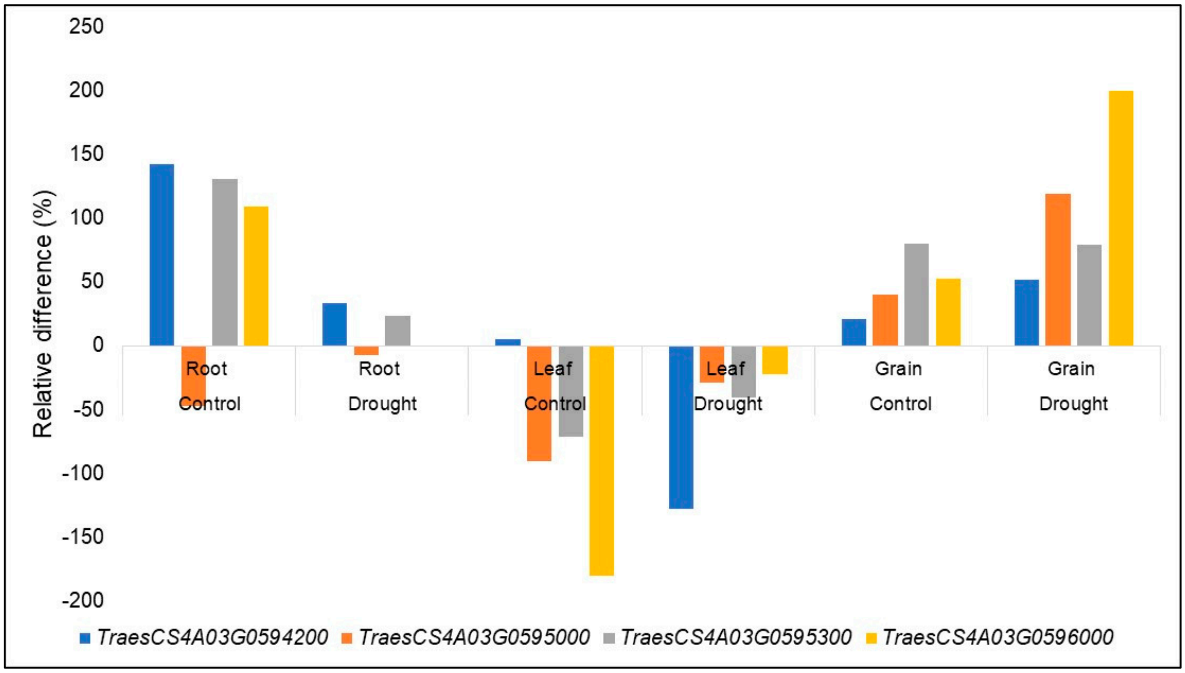

Figure S2. Relative percentage differences in gene expression of selected putative candidate genes across root, leaf, and grain tissues of resistant and susceptible varieties under control/well-watered and drought conditions. Data was retrieved from WheatOmics database.
