# Supplementary material for: Identification of SNPs and Candidate Genes Associated with Major Drought Tolerance QTL on Wheat Chromosome 4A
Source: Plants (Basel). 2026 Mar 16;15(6):921. doi: 10.3390/plants15060921 (PMC13029921; doi:10.3390/plants15060921)
Supplement: Supplementary file 1 [file plants-15-00921-s001.zip › Table S1.pdf]

plants

MDPI

Table S1. Candidate genes within 1 M bp in both upstream and downstream directions of SNP marker Kukri_c27037_112 near the target QTL on chromosome 4A.

No.	Candidate Genes	Start	End
1	*TraesCS4A03G0593200LC*	528302795	528303464
2	*TraesCS4A03G0593400*	528684125	528684385
3	*TraesCS4A03G0593500*	528685021	528687227
4	*TraesCS4A03G0593600*	528689873	528690323
5	*TraesCS4A03G0593700*	528691989	528692783
6	*TraesCS4A03G0593800LC*	528692865	528693434
7	*TraesCS4A03G0593900LC*	528872429	528872492
8	*TraesCS4A03G0594000LC*	529046437	529046622
9	*TraesCS4A03G0594100*	529176711	529178407
10	*TraesCS4A03G0594200*	529233709	529233891
11	*TraesCS4A03G0594300LC*	529236633	529237148
12	*TraesCS4A03G0594400*	529865945	529866262
13	*TraesCS4A03G0594500LC*	529876148	529876261
14	*TraesCS4A03G0594600*	529942358	529942415
15	*TraesCS4A03G0594700LC*	529945618	529945642
16	*TraesCS4A03G0594800LC*	529954345	529954692
17	*TraesCS4A03G0594900*	529986076	529986747
18	*TraesCS4A03G0595000*	529987389	529989560
19	*TraesCS4A03G0595100LC*	530121894	530121962
20	*TraesCS4A03G0595200*	530127329	530127329
21	*TraesCS4A03G0595300*	530127677	530127712
22	*TraesCS4A03G0595400LC*	530128176	530129210
23	*TraesCS4A03G0595500LC*	530130068	530131315
24	*TraesCS4A03G0595600LC*	530149079	530149182
25	*TraesCS4A03G0595700LC*	530153766	530154242
26	*TraesCS4A03G0595900*	530287216	530288367
27	*TraesCS4A03G0596000*	530297158	530297257
28	*TraesCS4A03G0596200LC*	530354311	530354619
29	*TraesCS4A03G0596300LC*	530355272	530355857
30	*TraesCS4A03G0596100*	530357988	530358732
31	*TraesCS4A03G0596400LC*	530359106	530359558
32	*TraesCS4A03G0596500*	530361281	530361534
33	*TraesCS4A03G0596600*	530375521	530375659
34	*TraesCS4A03G0596700LC*	530431075	530431617
35	*TraesCS4A03G0596800LC*	530431717	530431956
36	*TraesCS4A03G0596900*	530432968	530435709
37	*TraesCS4A03G0597000LC*	530723776	530724117
38	*TraesCS4A03G0597100*	530724118	530726856
39	*TraesCS4A03G0597200LC*	530736484	530736894
40	*TraesCS4A03G0597300LC*	530996555	530996795
