# Supplementary material for: Identification of SNPs and Candidate Genes Associated with Major Drought Tolerance QTL on Wheat Chromosome 4A
Source: Plants (Basel). 2026 Mar 16;15(6):921. doi: 10.3390/plants15060921 (PMC13029921; doi:10.3390/plants15060921)
Supplement: Supplementary file 1 [file plants-15-00921-s001.zip › Table S2.pdf]

plants

MDPI

Table S2. Tests of between-subjects for yield and thousand-grain weight (TGW) across genotypes (tolerant vs. susceptible) and environments (drought vs. control).

Source	Mean square		F		Significance		Partial Eta Squared η_p^2	
	Yield	TGW	Yield	TGW	Yield	TGW	Yield	TGW
Genotype	32.522	325.677	16.707	85.611	0.026	0.003	0.848	0.966
Environment	37.542	183.496	32.853	14.144	0.011	0.033	0.916	0.825
NILPair	13.327	138.284	4.58	9.008	0.068	0.033	0.735	0.876
Genotype * Environment	3.429	1.916	19.088	1.344	0.022	0.33	0.864	0.309
Genotype * NILPair	1.947	3.804	10.835	2.669	0.041	0.221	0.916	0.727
Environment * NILPair	1.143	12.973	6.361	9.101	0.081	0.051	0.864	0.901
Genotype * Environment * NILPair	0.18	1.426	0.501	0.707	0.684	0.555	0.045	0.062
**Simple effects of environment and genotype**								
**Environment**								
Control	7.415	138.817	20.684	68.856	< .001	<.001	0.393	0.683
Drought	28.536	188.777	79.604	93.637	< .001	<.001	0.713	0.745
**Genotype**								
Susceptible	31.832	111.457	88.798	55.285	<.001	<.001	0.735	0.633
Tolerant	9.139	73.956	25.494	36.684	<.001	<.001	0.443	0.534

Data analyzed using General Linear Model (GLM) Univariate analysis in SPSS v29.0, Significance at p <.005
