# Supplementary material for: Identification of SNPs and Candidate Genes Associated with Major Drought Tolerance QTL on Wheat Chromosome 4A
Source: Plants (Basel). 2026 Mar 16;15(6):921. doi: 10.3390/plants15060921 (PMC13029921; doi:10.3390/plants15060921)
Supplement: Supplementary file 1 [file plants-15-00921-s001.zip › Table S3.pdf]

plants MDPI

Table S3. Pairwise comparisons of yield and thousand-grain weight (TGW) of near-isogenic lines (NILs) under drought and control condition.

	(I) Genotype	(J) Genotype	Mean Difference (I-J)		Std. Error		Sig.[b]		95% Confidence Interval for Difference (Lower bound)		95% Confidence Interval for Difference (Upper bound)	
			Yield	TGW	Yield	TGW	Yield	TGW	Yield	TGW	Yield	TGW
**Environment**												
Control	Susceptible	Tolerant	-1.112*	-4.810*	0.24	0.6	<.001	<.001	-1.61	-5.99	-0.61	-3.63
	Tolerant	Susceptible	1.112*	4.810*	0.24	0.6	<.001	<.001	0.614	3.629	1.61	5.991
Drought	Susceptible	Tolerant	-2.181*	-5.609*	0.24	0.6	<.001	<.001	-2.68	-6.79	-1.68	-4.43
	Tolerant	Susceptible	2.181*	5.609*	0.24	0.6	<.001	<.001	1.683	4.428	2.679	6.79
**Genotype**												
Susceptible	Control	Drought	2.303*	4.310*	0.24	0.6	<.001	<.001	1.805	3.129	2.801	5.491
	Drought	Control	-2.303*	-4.310*	0.24	0.6	<.001	<.001	-2.8	-5.49	-1.81	-3.13
Tolerant	Control	Drought	1.234*	3.511*	0.24	0.6	<.001	<.001	0.736	2.33	1.732	4.692
	Drought	Control	-1.234*	-3.511*	0.24	0.6	<.001	<.001	-1.73	-4.69	-0.74	-2.33

Based on estimated marginal means. *The mean difference is significant at the .05 level. b Adjustment for multiple comparisons: Bonferroni.
